# Waist circumference is independently associated with liver steatosis and fibrosis in *LMNA*-related and unrelated Familial Partial Lipodystrophy women

**DOI:** 10.1186/s13098-023-01156-0

**Published:** 2023-09-07

**Authors:** Luiz F. Viola, Cynthia M. Valerio, João M. Araujo-Neto, Fabio F. Santos, Felipe Matsuura, Rodrigo O. Moreira, Amélio F. Godoy-Matos

**Affiliations:** 1 Instituto Estadual de Diabetes e Endocrinologia Luiz Capriglione (IEDE), Rua Moncorvo Filho 90, Rio de Janeiro, CEP: 20211-340 Brazil; 2 Federal University of São Paulo (UNIFESP), São Paulo, Brazil; 3 Faculdade de Medicina do Centro Universitário Presidente Antônio Carlos (FAME/UNIPAC), Juiz de Fora, Brazil; 4 Hospital Universitário Clementino Fraga Filho (HUCFF), Universidade Federal do Rio de Janeiro (UFRJ), Rio de Janeiro, Brazil

**Keywords:** Familial partial lipodystrophy, Non-alcoholic fatty liver disease, Liver fibrosis, Waist circumference

## Abstract

**Background:**

Lipodystrophies are a heterogeneous group of diseases characterized by the selective loss of subcutaneous adipose tissue and ectopic fat deposition in different organs, including the liver. This study aimed to determine the frequencies of liver steatosis (LS) and liver fibrosis (LF) in a sample of individuals with *LMNA*-related and unrelated Familial Partial Lipodystrophy.

**Methods:**

This cross-sectional study included 17 women with *LMNA*-related FPLD and 15 women with unrelated FPLD. LS and LF were assessed using transient elastography (TE) with FibroScan®. Anthropometric and biochemical variables were included in a multiple linear regression analysis to identify the variables that were independently related to liver disease.

**Results:**

Regarding the presence of LF, 22 (68.2%) women were classified as having non-significant fibrosis, and 10 (31.8%) were classified as having significant or severe fibrosis. Regarding LS, only six women (20.7%) were classified as having an absence of steatosis, and 23 (79.3%) had mild to severe steatosis. After multiple linear regression, waist circumference (but not age, body mass index, or waist-to-hip ratio) was found to be independently related to LS and LF. Among the biochemical variables, only triglyceride levels were independently related to LS but not LF.

**Conclusions:**

In women with FPLD, visceral fat accumulation appears to be the most important determinant of liver disease, including LF, rather than fat scarcity in the lower limbs.

## Highlights

Familial Partial Lipodystrophy (FPLD) is a group of heterogeneous diseases characterized by selective loss of subcutaneous adipose tissue.

Non-alcoholic fatty liver disease (NAFLD) is an important complication of FPLD that can develop early in the course of disease and may progress to end stage liver disease.

Transient Elastography (TE) with FibroScan is a noninvasive method that can provide useful information on both the degree of liver steatosis (LS) and fibrosis (LF).

Our study uses TE to determine the frequency of LS and LF in individuals with FPLD, and to determine which anthropometric/biochemical variables would correlate with LS and LF.

Our findings suggest that waist circumference was a significant parameter that independently correlates with both LF and LS. These findings reinforce the role of excess visceral fat in addition to fat loss in the lower limbs in the severity of NAFLD in patients with FPLD.

## Introduction

Familial Partial Lipodystrophy (FPLD) is a group of heterogeneous diseases characterized by selective loss of subcutaneous adipose tissue and ectopic fat deposition in the liver, muscle, pancreas, and other organs, and are associated with metabolic repercussions of insulin resistance (IR), such as diabetes mellitus (DM), hypertriglyceridemia, and non-alcoholic fatty liver disease (NAFLD). Traditionally, they are classified according to the extent of body fat loss (partial or generalized) and etiology (genetic or acquired) [1–3]. Among familial variants of FPLD, the most common subtype is the Dunnigan variety (FPLD type 2; FPLD2), caused by heterozygous mutations in the *LMNA* gene [1, 2]. In contrast, no disease-causing mutations have been identified in the Köbberling phenotype (FPLD type 1; FPLD1), suggesting a polygenic contribution [4].

Partial lipodystrophies usually present with the absence of fat in the limbs, hips, and buttocks, along with deposition of excess fat in the cervical, facial, and intra-abdominal regions. The lack of fat develops during childhood and/or puberty [2–5], and these individuals usually have a high cardiovascular risk [6]. Specifically, Köbberling’s lipodystrophy is a variant that affects the forearm, calves, and/or, more rarely, the entire limbs, and is associated with the presence of DM, prediabetes and acanthosis nigricans. Patients with Köbberling lipodystrophy may present a more severe metabolic profile than that observed in FPLD2 patients [7].

As expected, a background of IR and reduced ability to store triglycerides may predispose patients with lipodystrophy to a high prevalence of NAFLD [8]. Indeed, generalized forms of hepatic steatosis have been described in younger patients (median age at diagnosis 12 years), but few studies have characterized the real prevalence and severity of NAFLD in patients with FPLD [9, 10]. Given the worldwide prevalence of NAFLD, medical guidelines have been published to guide the screening and management of individuals at risk of NAFLD. In this context, liver transient elastography (TE) is a non-invasive method for diagnosing NAFLD and liver fibrosis [11]. Given the great heterogeneity of partial lipodystrophy patients and the lack of studies describing the real prevalence of NAFLD in FPLD patients, the aims of this study were to evaluate the occurrence of liver steatosis (LS) and liver fibrosis (LF) in patients with FPLD using TE and to correlate these findings with metabolic and anthropometric parameters in this population.

## Patients and methods

### Patients

Thirty-two women with FPLD were sequentially selected from the outpatient clinics of the Metabolism Unit of the State Institute of Diabetes and Endocrinology (Rio de Janeiro, Brazil) from January 2020 to June 2021. Clinical diagnosis of FPLD phenotype was determined using criteria from previous reports [1, 2]. All individuals presented at least 3 of the following features: postpubertal loss of adipose tissue affecting the lower limbs while sparing the face and neck (essential criteria), prominent veins and muscularity, acanthosis nigricans, polycystic ovarian syndrome (PCOS), hypertriglyceridemia and/or low high-density-lipoprotein (HDL) cholesterol, steatohepatitis, DM or impaired fasting glucose (IFG), and similar fat distribution and/or history of fat loss in a first-degree relative.

Deficiency of subcutaneous body fat (an essential criterion) was considered after physical examination by two experienced physicians and confirmed with low skinfold thickness in the anterior thigh according to caliper measurements in men (≤ 10 mm) and women (≤ 22 mm). PCOS was defined according to the Rotterdam criteria with no other known causes. DM was identified as two fasting glucose measurements ≥ 126 mg/dl or use of antidiabetic agents, and glucose intolerance was characterized as fasting glucose ≥ 100 mg/dl (IGF) or glucose ≥ 140 mg/dl after the oral glucose tolerance test. Hypertriglyceridemia was diagnosed with triglycerides ≥ 150 mg/dl, and low HDL-cholesterol level was defined as < 50 mg/dl.

Exclusion criteria were age under 18 years, pregnancy or breastfeeding, presence of acquired forms of lipodystrophy (autoimmune or related to HIV infection or use of highly active antiretroviral therapy), and severe renal disease (estimated glomerular filtration rate less than 15mL/min/m^2^). The patients in this study had no evidence of other forms of liver disease and had alcohol consumption of < 40 g/week.

FPLD-negative patients showed no mutations in the *LMNA* gene, but were clinically diagnosed with FPLD. The search for variants in 28 genes involved in the aetiology of congenital lipodystrophies was first performed by NGS (Ion Torrent System, Thermo Fisher Scientific, Waltham, MA, USA) sequencing of the entire coding region of the genes and the flanking intronic regions *ABCA1 AGPAT2 AKT2 APOA5 APOC2 BSCL2 CAV1 CAVIN1 CFTR CIDEC CTRC CYP27A1 GPIHBP1 LIPA LIPE LMF1 LMNA LMNB2 LPL MFN2 PLIN1 POLD1 PPARG PRSS1 PSMB8 SMPD1 SPINK1 ZMPST*). *LMNA*-related FPLD diagnosis was confirmed by molecular analysis of the LMNA gene – Sanger sequencing (ABI Prism 3100 Genetic Analyzer; Applied Biosystems, Foster City, CA, USA).

The study protocol was approved by the local ethics committee, and all of the subjects gave their written informed consent. Each patient underwent a physical examination was, and previous medical records were analysed to verify the historical details.

### Anthropometric measures

The following anthropometric data were recorded from all participants: body weight (kg), height (m), waist circumference, waist-to-hip ratio (WHR), and blood pressure. Body mass index (BMI) was calculated as weight in kilograms divided by the square of height in meters (kg/m^2^). Waist circumference was determined at the midpoint between the lowest rib and iliac crest. WHR was defined as the ratio of the waist girth to the largest circumference of the hip, measured at the greater trochanter. Skin thickness was assessed using standardized techniques with a Lange caliper (Cambridge Scientific Industries, Cambridge, MD, USA) on the triceps and the anterior thigh on the right side of the body. The mean of three repeated measurements was calculated for each site [12].

### Laboratory evaluation

Blood samples were collected between 6:30 am and 8:00 am after overnight fasting (12 h). Plasma glucose levels were determined using the glucose-oxidase method. The cholesterol content of the lipoprotein fractions and triglycerides were measured enzymatically. Plasma leptin levels were determined by enzyme-linked immunosorbent assay (ELISA).

### Transient elastography (TE)

Transient elastography was performed using FibroScan®. Participants were examined at the University Hospital of the Federal University of Rio de Janeiro, Brazil. The examinations were performed by a single experienced physician after four hours of fasting. At least ten measurements were obtained with the patient in the supine position.

Steatosis was quantified using the controlled attenuation parameter (CAP), and the following cut-offs were applied: CAP ≤ 248 dB/m: S0—steatosis absent; 248 < CAP ≤ 268 dB/m: S1—mild steatosis; 268 < CAP ≤ 280 dB/m: S2—moderate steatosis; CAP ≥ 280 dB/m: S3—advanced steatosis. Fibrosis was classified as follows: LSM ≤ 6.9 kPa: F0/F1—absent or minimal fibrosis; 6.9 < LSM < 8.4: F2—moderate fibrosis; LSM ≥ 8.4: F3/F4—advanced fibrosis.

### Statistical analysis

Statistical analysis was performed using GraphPad InStat 3.00 for Windows 95 (GraphPad Software, San Diego, CA, USA). Parametric data are presented as mean ± standard deviation (SD), and nonparametric data are presented as median (range, minimum-maximum). Unpaired *t*-tests were used to compare parametric variables, and the Mann–Whitney test was used for nonparametric variables. The strength of the linear relationship between the two continuous variables was evaluated using Pearson’s correlation coefficient or Spearman’s correlation coefficient. Multiple linear regression was used to identify the variables independently related to liver steatosis (CAP) and liver stiffness (LS). The level of statistical significance was 5%.

## Results

A total of 32 women were included (17 with *LMNA*-related and 15 with unrelated FPLD). The mean age of the sample was 49.1 ± 11.7 years old; the median BMI was 25.5 kg/m^2^ (range, 19.9–39.1); mean WC and hip circumference were 95.2 ± 14.6 cm and 96.0 ± 14.5 cm, respectively; and median WHR was 0.96 (range, 0.85–1.70). The individuals with *LMNA*-related FPLD belonged to eight families. Twelve women were heterozygous for c.1444 C > T variant p.R482W, three individuals harbored c.1445G > A p.R482Q, and one harbored the heterozygous variant c.1396AOG p.N466D, all in exon 8. One woman had a heterozygous variation c.1744 C > T p.R582C in exon 11. Some of these variants have been previously reported [13, 14].

Fifteen women were included in the *LMNA* unrelated FPLD subgroup of this study. They presented with limb lipoatrophy, central obesity, and increased facial, neck, and abdominal adiposity, with a ledge between the affected (lipodystrophic) and non-affected areas, and metabolic complications related to severe IR. Most patients presented with DM (n = 12), hypertriglyceridemia, and acanthosis. None of them showed mutations in *LMNA* gene.

Quantification of steatosis revealed that six individuals (20.7%) had no steatosis (S0), two individuals (6.9%) had mild steatosis (S1), one individual (3.4%) had moderate steatosis (S2), and 20 individuals (68.9%) had severe steatosis (S3). Data from 3 patients were missing. Fibrosis evaluations showed 22 (68.2%) individuals with nonsignificant fibrosis (F0/F1), three individuals (9.4%) with moderate fibrosis (F2), and seven individuals (21.8%) with advanced fibrosis (F3/F4).

**Fig. 1:**
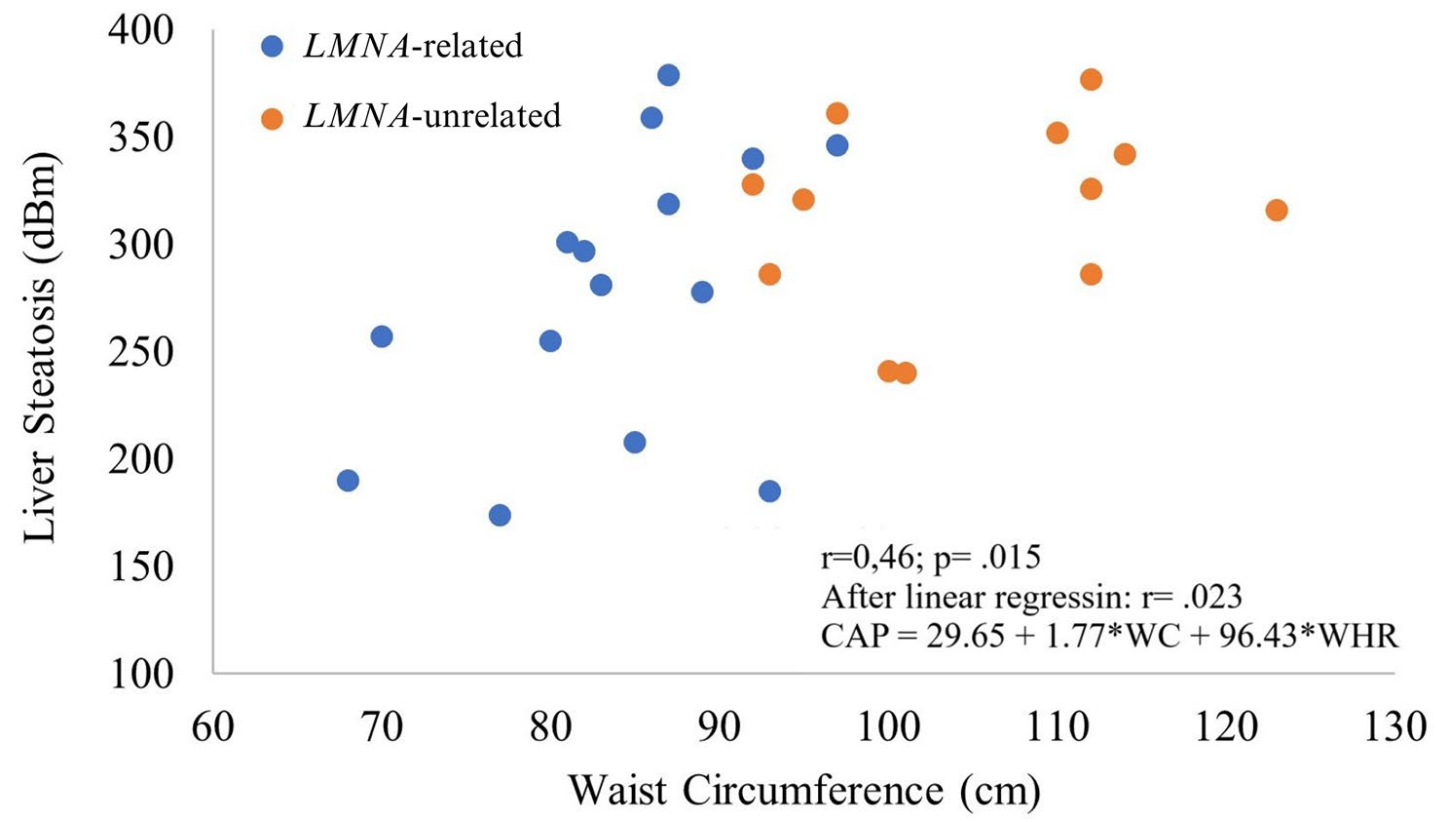
Correlation between waist circumference and liver steatosis measured by Transient Elastography in a sample of individuals with *LMNA*-related and unrelated Familial Partial Lipodystrophy; *r* = .46; *P* = .015 for liver steatosis

Correlation analysis was used to determine the anthropometric parameters associated with liver steatosis. Data were not available for five individuals. A positive correlation was found between CAP and both WC (*r* = .46; *P* = .015; Fig. 1) and WHR (*r* = .52; *P* = .0056). In contrast, no correlation was found between CAP and age (*r* = − .01; *P* = .93), BMI (*r* = .27; *P* = .14), or hip circumference (*r* = .17; *P* = .38). After multiple linear regression analysis, only WC remained independently related to CAP (*P* = .023).

**Fig. 2:**
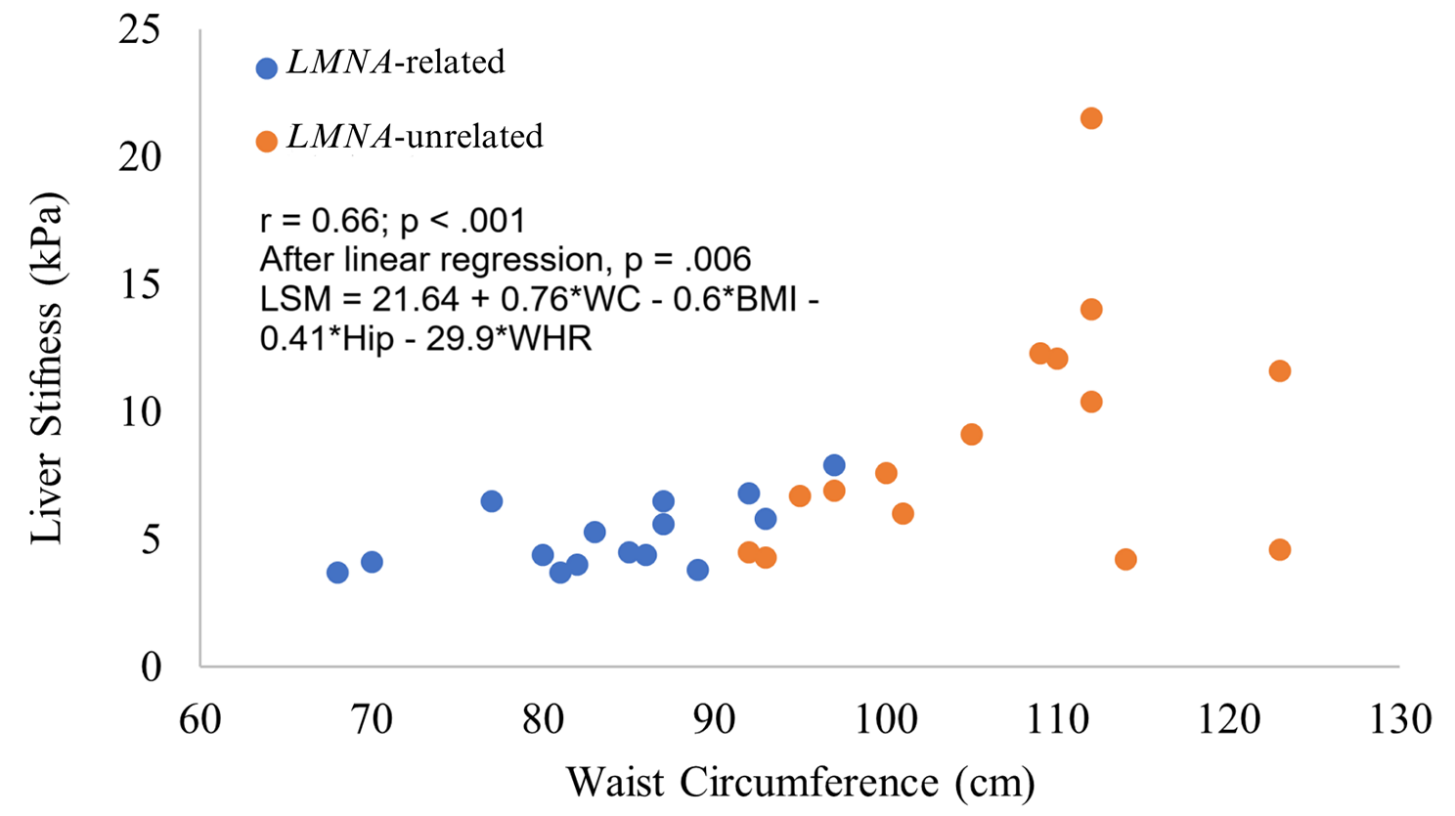
Correlation between waist circumference and liver stiffness measured by Transient Elastography in a sample of individuals with *LMNA*-related and unrelated Familial Partial Lipodystrophy; *r* = .66; *P* < .001 for liver stiffness

The same analysis was conducted for patients with liver fibrosis. Data were not available for two individuals. A positive correlation was found between LSM and BMI (*r* = .47; *P* = .0067), WC (*r* = .66; *P* < .001; Fig. 2), hip circumference (*r* = .46; *P* = .0094), and WHR (*r* = .52; *P* = .0038). A borderline significant relationship was found between LSM and age (*r* = .34; *P* = .05). After multiple linear regression, only WC remained independently related to LSM (*P* = .006).

Biochemical variables were also analyzed to determine whether laboratory parameters were associated with liver steatosis and fibrosis. Regarding steatosis, no correlation was found between CAP and leptin (*r* = .25; *P* = .20) or HDL cholesterol (*r* = − .15; *P* = .43). However, a positive correlation was found between CAP and triglycerides (*r* = .39; *P* = .036). In multiple linear regressions, both WC and triglycerides, as independent variables, were independently associated with liver steatosis (*P* = .01, WC; *P* = .045, triglycerides). Regarding fibrosis, a significant correlation was found between liver stiffness and both leptin (*r* = .39; *P* = .036) and HDL cholesterol (r = − .35; P = .043) but not with triglycerides (*r* = .19; *P* = .28). In a multiple linear regression analysis using WC, leptin, and triglycerides as independent variables, only WC remained independently related to liver stiffness (*P* = .006).

## Discussion

Disruptions in body fat distribution are frequently associated with metabolic complications including DM and cardiovascular diseases. However, few studies have investigated the association between lipodystrophic syndromes and NAFLD. In this study, the frequencies of LS and LF in a sample of women with FPLD were determined using TE. Among the study population, 80% had LS and approximately 30% had significant fibrosis. In comparison, the global prevalence of NAFLD is 25% [15]. We also investigated which anthropometric and/or biochemical findings were independently associated with liver disease. Interestingly, WC was the only independent predictor of LS and LF in this population.

The full spectrum of NAFLD (hepatic steatosis, non-alcoholic steatohepatitis [NASH], NAFLD fibrosis, cirrhosis, and hepatocellular carcinoma) has already been partially described in lipodystrophy patients [16]. Interestingly, most recent guidelines suggest that NASH diagnosis in a lean patient should raise suspicion of lipodystrophy [1]. The Endocrine Society Guideline for Diagnosis and Management of Lipodystrophy Syndromes recommends ultrasonography and aminotransferase measurement annually for all patients with lipodystrophy, with liver biopsy as clinically indicated [1]. This could be considered as the first challenge in diagnosing NAFLD in FPLD patients. Even though FPLD patients are a patient subgroup at high risk for NASH and liver fibrosis, routine screening with liver biopsy seems unfeasible for all patients with lipodystrophy-like phenotype. In this context, the use of TE, a noninvasive diagnostic method for assessing the degree of liver stiffness, seems reasonable and may be useful as a screening test to reduce the need for liver biopsy [17–19].

Some case series have described the frequency and severity of NAFLD in patients with FPLD. In a descriptive analysis of a trial cohort of 23 individuals with partial lipodystrophy (seven with FPLD type 2), Ajluni et al. [20] found a high prevalence of NASH with fibrosis; 22 patients met the criteria for NASH via liver biopsy, and 18 patients (78.3%) had some degree of fibrosis with variable severity. They also found a positive correlation between liver fat (quantified using the Dixon method) and both HbA1c and log-transformed triglyceride levels (20). In comparison, we observed a lower prevalence of hepatic fibrosis (31.8%), as evaluated using TE. After multiple linear regression analysis using WC, leptin, and triglycerides as independent variables, only WC remained independently related to liver stiffness *(P =* .006). Notably, our patients were leaner (median BMI 25.5 versus 27.3 kg/m^2^) and presented a lower WHR (0.96 versus 0.99), with less metabolic derangements, as compared to this trial cohort.

Although the current data cannot prove causality, our study reinforces the importance of central fat distribution in NAFLD pathogenesis in a FPLD population. A recent meta-analysis by Pang et al. investigated the association between central and general obesity and NAFLD in a non-lipodystrophy population. They found that WC was a better predictor for NAFLD than BMI, although both were associated with an increased risk of NAFLD [21]. In another study, in a non-lipodystrophy population of patients with obesity or overweight, both WC and BMI were independently associated with CAP score, although WC was a better predictor [22]. A review by Kechagias et al. discussed the prevalence of NAFLD in lean patients with BMI < 25 kg/m^2^ but with excess visceral fat, noting that this appears to be a distinct group of patients with a particular pathophysiology, including genetics [15]. Finally, another recent analysis, performed in 121 patients with DM type 1, found that visceral adipose tissue, but not peripheral body fat, was associated with NAFLD [23]. Altogether, these findings reinforce the importance of visceral adipose tissue in the pathogenesis of NAFLD.

Conflicting evidence exists regarding how visceral fat affects metabolic disruption in patients with lipodystrophy. Guillín-Amarelle et al. [7] compared metabolic derangements and body composition in 98 patients with FLPD1 and 25 patients with FPLD2. They demonstrated that metabolic disease was associated with visceral fat and inversely related to lower extremity fatness in patients with FPLD. Similar to our results, the prevalence of hypertriglyceridemia and NAFLD (assessed by ultrasonography) was higher in the LMNA-negative group (66.3% and 77.6%, respectively) than in the *LMNA*-linked group. In contrast, Malandrino et al. [24] did not find a relationship between visceral fat distribution and metabolic diseases in patients with lipodystrophy. Moreover, they hypothesized that visceral fat distribution was not a major contributor to metabolic diseases. However, most of the included patients used antidiabetic or lipid-lowering medications. We believe that the inclusion of lean FPLD (mean BMI 25.7 kg/m2) and patients with generalized lipodystrophy in their study may be an interesting explanation for these divergent results.

Additionally, Lotta et al. [4] found that the polygenic contribution might be associated with lower body fat in patients with type 1 FPLD. This may be relevant for investigating the genetic drivers of body fat distribution and metabolic derangement in patients with multiple types of lipodystrophies.

Our study has several limitations. First, a liver biopsy was not performed to confirm liver steatosis or fibrosis. However, the use of TE with a controlled attenuation parameter has demonstrated good accuracy in quantifying the degrees of liver steatosis and fibrosis [19]. Second, body composition was assessed using anthropometric and skinfold parameters. Other imaging methods (e.g., DXA) can provide more detailed information. In contrast, WC is widely used in clinical practice and is well-accepted as a reliable measurement of visceral fat. Moreover, it has recently been demonstrated in individuals with lipodystrophy that WC is associated with IFN-γ and IL-6 [25], which are cytokines that have been implicated in the pathogenesis of NAFLD [15]. Third, not all patients had leptin measurements available.

Our data suggests that NAFLD is highly prevalent in the FPLD population. This reinforces the importance of early screening of affected patients. The strength of this study was the use of a non-invasive method to screen for both LS and LF. However, further studies evaluating patients with FPLD should be conducted to validate this tool.

In conclusion, it seems plausible to consider that among the predominant mechanisms described in the development of NAFLD in FPLD patients, visceral fat accumulation might be an important driver of disease progression.

## Data Availability

The datasets used and/or analyzed during the current study are available from the corresponding author upon reasonable request.

## Abbreviations

IR: Insulin resistance
FPLD: Familial partial lipodystrophies
DM: Diabetes mellitus
NAFLD: Non-alcoholic fatty liver disease
TE: Transient elastography
LS: Liver steatosis
LF: Liver Fibrosis
PCOS: Polycystic ovarian syndrome
(HDL) cholesterol: High-density-lipoprotein
IFG: Impaired fasting glucose
HIV: Human Immunodeficiency Virus
WHR: Waist-to-hip ratio
CAP: Controlled attenuation parameter
LSM: Liver stiffness measurement
WC: Waist circumference
BMI: Body Mass Index
NASH: Non-alcoholic steatohepatitis
DXA: Dual-energy X-ray absorptiometry
